# Rapid iPSC-derived neuromuscular junction model uncovers motor neuron dominance in amyotrophic lateral sclerosis cytopathy

**DOI:** 10.1038/s41420-025-02302-5

**Published:** 2025-01-25

**Authors:** Hsiao-Chien Ting, Yun-Ting Guo, Hong-Lin Su, Yu-Shuan Chen, Shinn-Zong Lin, Horng-Jyh Harn, Chia-Yu Chang

**Affiliations:** 1 Bioinnovation Center, Buddhist Tzu Chi Medical Foundation, Hualien, Taiwan; 2 Department of Life Sciences, National Chung Hsing University, Taichung, Taiwan; 3 The iEGG and Animal Biotechnology Research Center, National Chung Hsing University, Taichung, Taiwan; 4 Department of Medical Research, Hualien Tzu Chi Hospital, Buddhist Tzu Chi Medical Foundation, Hualien, Taiwan; 5 Neuroscience Center, Hualien Tzu Chi Hospital, Buddhist Tzu Chi Medical Foundation, Hualien, Taiwan; 6 Center for General Education, Tzu Chi University, Hualien, Taiwan; 7 Department of Neurosurgery, Hualien Tzu Chi Hospital, Buddhist Tzu Chi Medical Foundation, Hualien, Taiwan; 8 Department of Pathology, Hualien Tzu Chi Hospital and Tzu Chi University, Hualien, Taiwan

**Keywords:** Amyotrophic lateral sclerosis, Neuromuscular junction, Stem-cell research

## Abstract

The neuromuscular junction (NMJ) is essential for transmitting signals from motor neurons (MNs) to skeletal muscles (SKMs), and its dysfunction can lead to severe motor disorders. However, our understanding of the NMJ is limited by the absence of accurate human models. Although human induced pluripotent stem cell (iPSC)-derived models have advanced NMJ research, their application is constrained by challenges such as limited differentiation efficiency, lengthy generation times, and cryopreservation difficulties. To overcome these limitations, we developed a rapid human NMJ model using cryopreserved MNs and SKMs derived from iPSCs. Within 12 days of coculture, we successfully recreated NMJ-specific connectivity that closely mirrors in vivo synapse formation. Using this model, we investigated amyotrophic lateral sclerosis (ALS) and replicated ALS-specific NMJ cytopathies with SOD1 mutant and corrected isogenic iPSC lines. Quantitative analysis of 3D confocal microscopy images revealed a critical role of MNs in initiating ALS-related NMJ cytopathies, characterized by alterations in the volume, number, intensity, and distribution of acetylcholine receptors, ultimately leading to impaired muscle contractions. Our rapid and precise in vitro NMJ model offers significant potential for advancing research on NMJ physiology and pathology, as well as for developing treatments for NMJ-related diseases.

## Introduction

Neuromuscular junctions (NMJs) are critical sites where motor neurons (MNs) transmit acetylcholine to skeletal muscles (SKMs), thereby triggering contractions to initiate movement in humans [1]. NMJ dysfunction leads to severe motor disorders, such as amyotrophic lateral sclerosis (ALS) [2] and spinal muscular atrophy [3].

ALS is a progressive motor function disorder affecting 1–2 people per 100,000. Most patients lose motor function and die within 3–5 years [4]. Current treatments only partially alleviate the late stages of the disease but are not curative [5–7]. Despite extensive efforts in developing drugs targeting neuronal pathologies, most have been ineffective [8, 9]. Therefore, developing effective early-stage ALS treatments is a critical focus of pharmacological research. NMJs are the first tissues to exhibit ALS-specific pathology, thereby directly impairing motor function in patients [10–12]. This emphasizes the importance of NMJ research in the development of early ALS treatments. However, understanding the interactions and their disruptions of NMJ function in diseases is challenging because of the lack of precise human NMJ models. Existing models based on rodents, cell lines, and primary cells differ significantly from human NMJs [13–15], highlighting the need to establish accurate human models. Furthermore, few studies have explored the mechanisms and pathological roles of MNs and SKMs in human NMJ models of ALS.

Human induced pluripotent stem cells (iPSCs), which can self-renew and differentiate into various cell types, present a promising solution for developing accurate patient-derived NMJ models [16]. iPSC-derived NMJ models and organoids can establish NMJ-like connections and have been used to explore disease mechanisms and improve current understanding of human NMJs [17–32]. However, most of these models are constrained by the need for transgene overexpression, long-term cocultures or organoid formation, and difficulties in handling and cryopreservation. Establishing NMJ models using iPSCs requires a continuous, uninterrupted period of at least 24–35 days, even with specific gene overexpression [23–26]. Without exogenous gene expression, the timeframe extends to 35–47 days in coculture models [18–20, 22, 28] and 50–100 days in self-organized organoids [29–31]. A simpler, shorter-term, and more user-friendly human NMJ model could help overcome these challenges, significantly advance research on NMJ-related diseases, and facilitate drug development for neuronal conditions like ALS.

In this study, we developed human NMJ-like tissue within a continuous 12-day period using cryopreserved MNs and SKMs derived from iPSCs. These tissues exhibited NMJ structures, expressed acetylcholine receptors (AChRs), and demonstrated inducible SKM contractions. For ALS modeling, we generated NMJs using two pairs of SOD1 mutant ALS and isogenic healthy iPSC lines. Our results revealed ALS-specific cytopathies in our NMJ model that closely resemble those observed in patient tissues. Interestingly, we found that SOD1 MNs significantly reduced AChR expression, exhibited altered AChR properties, and impaired myotube contractions, demonstrating their dominant role in initiating NMJ cytopathies in ALS. Our human NMJ model provides a simple, short-term, and effective platform for studying MN–SKM interactions and exhibits potential utility for large-scale drug screening in neuromuscular diseases.

## Results

### Robust generation of ready-to-use cryopreserved MN stocks

We developed the CHSF–MN protocol, a highly efficient MN differentiation method for generating ready-to-use cryopreserved MN stocks. This protocol converted iPSCs into 90% HB9^+^ MNs, followed by a high-yield MN cryopreservation technique, as previously reported [33].

**Fig. 1:**
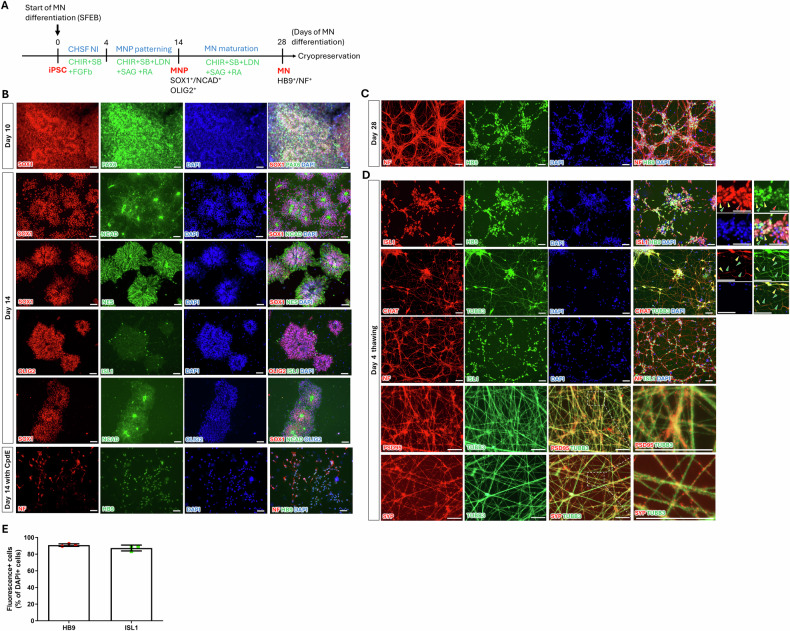
Robust generation of ready-to-use cryopreserved motor neurons (MNs) from induced pluripotent stem cells (iPSCs). **A** Procedure of MN differentiation and cryopreservation. **B** Immunocytochemistry (ICC) staining of differentiated cells for neural stem cell (NSC) markers PAX6, SOX1, NES and NCAD; motor neuron progenitor (MNP) marker OLIG2; and MN marker ISL1 and HB9 in differentiated cells on day 10 and day 14. **C** ICC for MN markers HB9 and NF on day 28 of differentiation. **D** ICC for MN markers HB9, ISL1, CHAT, and neuronal marker NF, PSD95 and SYP on day 4 of thawing. Green arrowheads indicate the green fluorescence cells, red arrowheads indicate the red fluorescence cells, and yellow arrowheads indicate cells positive for both green and red fluorescence. **E** Calculation of the expression ratio of relevant markers in DAPI^+^ cells on day 4 post-thaw. 6 images from 3 independent experimental replicates were analyzed, with each data point representing the mean value from a single replicate. The procedure in (**A**) was generated using Microsoft PowerPoint. SFEB serum-free embryoid body, NI neural induction, CHIR CHIR99021, SB SB431542, FGFb FGF-basic, LDN LDN193189, CpdE Compound E, and RA retinoic acid. Scale bars: 50 μm (**B**, **C**, **D**).

In this study, the SOD1^G85G^ healthy iPSC line, which was corrected from the SOD1^G85R^ mutant iPSC line, was first used for MN differentiation [34], followed by validation in a second isogenic pair [35]. The MN differentiation process is briefly outlined in Fig. 1A. On day 10 of differentiation, neural stem cell (NSC) markers PAX6 and SOX1 were highly co-expressed. On day 14, NSC and motor neuron progenitor (MNP) markers, including SOX1, N-cadherin (NCAD), Nestin (NES), and OLIG2, were highly expressed (Fig. 1B). Moreover, the high co-expression of the NSC marker SOX1, along with NCAD and the MNP marker OLIG2, indicated that the majority of NSCs were MNPs. At this stage, the MN-specific marker ISL1 began to be expressed. With the stimulation of Compound E (a chemical that accelerates neuronal maturation) [36], the MN-specific transcription factor HB9 was expressed, but the nerve fiber marker Neurofilament (NF) was absent, indicating MN fate determination had occurred, although the cells had not yet fully matured into MNs. By day 28, most of the cells were expressing NF and HB9 (Fig. 1C), confirming that the cells at this stage were MNs. MN spheres were cryopreserved on the same day. After 4 days of thawing (32 days of differentiation in total), the cells robustly expressed classical neuronal markers NF and β-III-tubulin (TUBB3), synaptic markers PSD95 and Synaptophysin (SYP), as well as MN-specific markers HB9, ISL1, and Choline O-acetyltransferase (CHAT) (Fig. 1D). Green arrowheads indicate green fluorescence-labeled cells, red arrowheads indicate red fluorescence-labeled cells, and yellow arrowheads indicate double-positive cells in Fig. 1D. Statistical analysis showed that 90.9% ± 0.87% of the differentiated cells expressed HB9, and 87.38% ± 2.04% expressed ISL1 four days after thawing (Fig. 1E). These results confirmed that the thawed cells were high purity MNs. Undifferentiated iPSCs were used as negative controls for SOX1, NCAD, NF, TUBB3, PAX6, NES, PSD95, and SYP, while non-motor neurons were used as negative controls for OLIG2, ISL1, HB9, and CHAT. As shown in Fig. S1, all fluorescence signals for these primary antibodies were negative in the controls, demonstrating the specificity of the antibodies.

### Differentiation of iPSCs into skeletal myoblast stocks and further SKM maturation

**Fig. 2:**
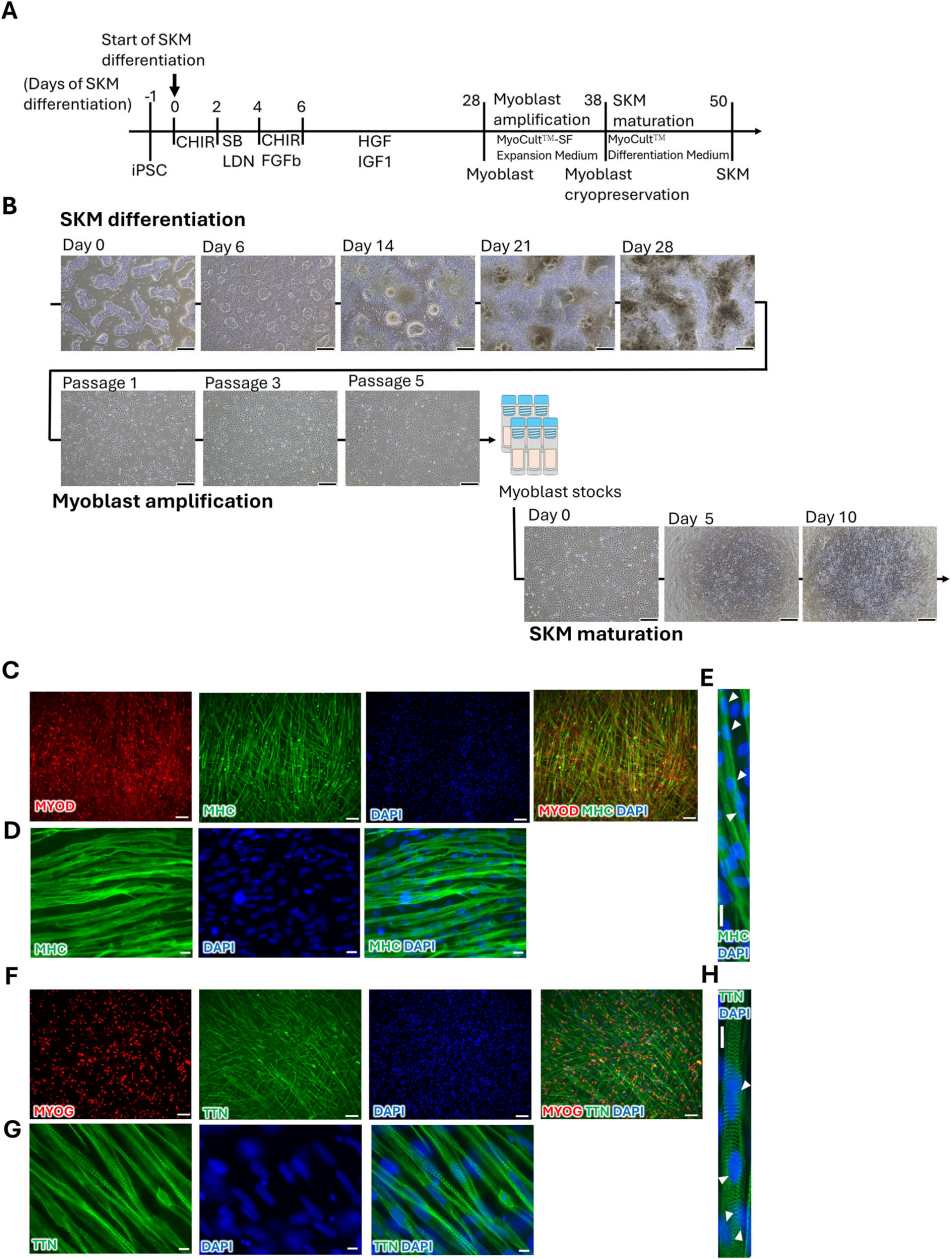
Generation of myoblast cryopreservation stocks and further skeletal muscle (SKM) maturation from induced pluripotent stem cells (iPSCs). **A** Differentiation procedure of skeletal muscles (SKM). **B** Cell morphology during SKM differentiation, amplification, and maturation. **C**–**H** Immunocytochemistry (ICC) of differentiated cells for SKM markers MHC, MYOD, MYOG, and TTN on day 12 of SKM maturation; Arrowheads indicate the multinuclear morphology in (**E**, **H**). The procedure in (**A**) was generated using Microsoft PowerPoint. Scale bars: 300 μm (**B**); 100 μm (**C**, **F**); 20 μm (**D**, **E**, **G**, **H**).

To obtain high-purity human SKMs, we applied a transgene-free protocol for SKM differentiation, as described previously [37] with modifications (Fig. 2A). The morphological transition from undifferentiated iPSCs to SKMs was shown in Fig. 2B. After 28 days of differentiation, skeletal myoblast-like cells were dissociated and amplified for five passages (10 days) before cryopreservation. These cryopreserved myoblast stocks from passage 5 were used for subsequent experiments. The skeletal myoblasts exhibited myotube-like spindle structures on day 12 of terminal SKM maturation after thawing. Immunocytochemical (ICC) analysis confirmed the expression of SKM-specific markers, including Myogenic Differentiation 1 (MYOD), Myogenin (MYOG), Myosin Heavy Chain (MHC), and Titin (TTN) (Fig. 2C–H). Furthermore, the cells displayed typical SKM characteristics, such as multinucleation (indicated by arrowheads), cross-striation, and TTN fiber banding (Fig. 2E, H). Thus, the differentiation, expansion, and cryopreservation of skeletal myoblasts and maturation of SKMs were successfully established.

### Establishment of NMJ-like tissue within 12 days of coculture

**Fig. 3:**
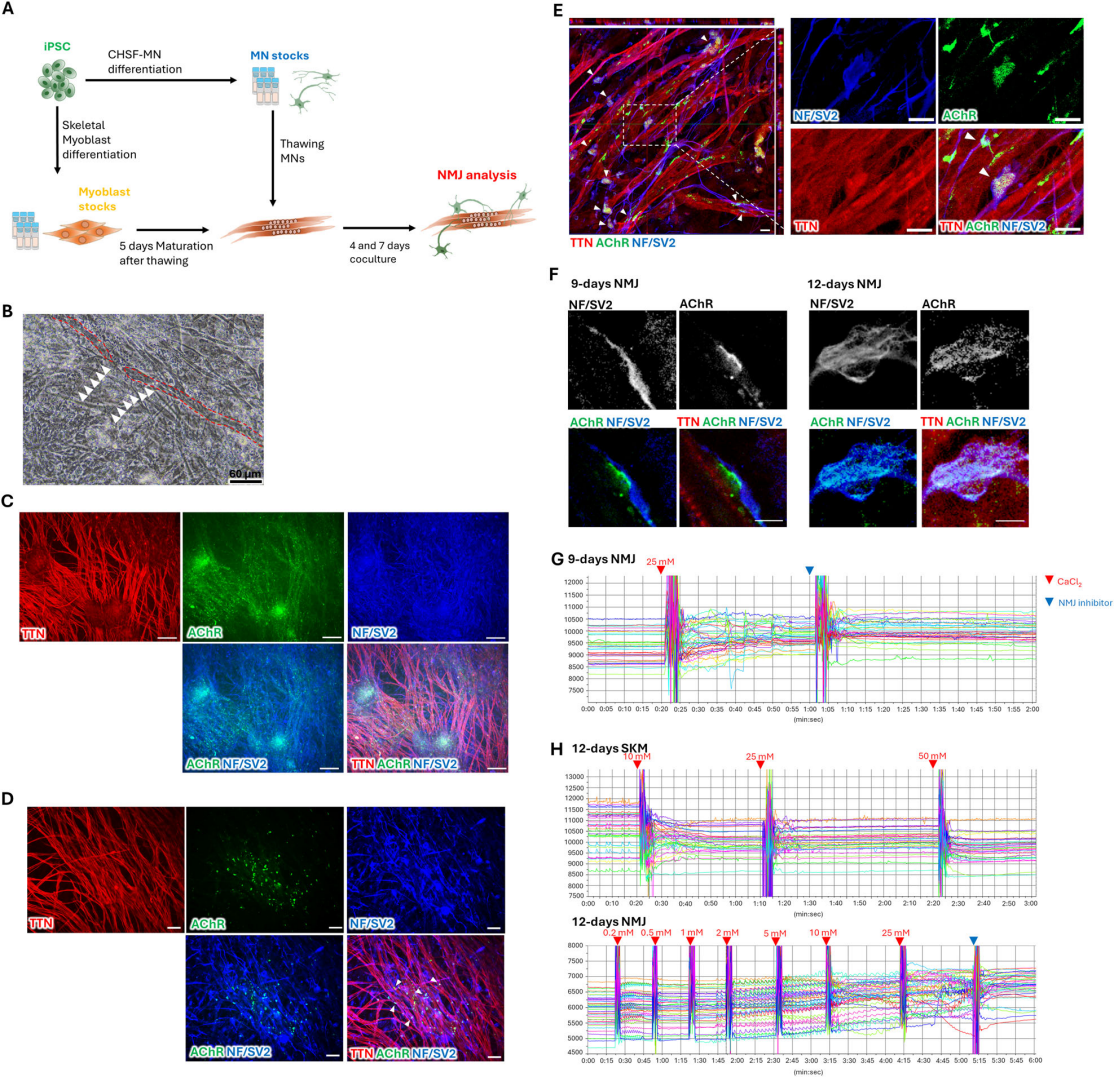
Neuromuscular junction (NMJ) model derived from the coculture of thawed skeletal muscle (SKM) and motor neurons (MN). **A** Establishment of NMJ model from SKM–MN coculture. **B** Cell morphology of day 9 SKM–MN coculture. Arrowheads indicate the neurites and red dot area indicate the myotubes. **C**, **D** Immunocytochemistry (ICC) staining for SKM marker TTN; neuronal marker NF; and NMJ markers SV2 and AChR. Arrowheads indicate the regions of overlap between AChR, NF/SV2, and TTN. **E** Confocal image showing the colocalization of TTN, NF/SV2, and AChR. Arrowheads indicate the colocalization of AChR, NF/SV2 and TTN. **F** Confocal image showing the colocalization of TTN, NF/SV2, and AChR on day 9 and day 12 NMJs. **G**, **H** Bright-field intensity changes in different regions of interest after CaCl_2_ induction and NMJ inhibitor treatment on day 9 NMJ (**G**), day 12 SKM (**H**) and day 12 NMJ (**H**). The procedure in (**A**) was generated using Microsoft PowerPoint. Scale bars: 60 μm (**B**); 100 μm (**C**); 30 μm (**D**); 20 μm (**E**) and 5 μm (**F**).

To establish the rapid human iPSC-derived NMJ model, myoblasts were thawed and matured into SKMs over the first 5 days. Ready-to-use MN spheres were then thawed and seeded directly onto the myotube layer. The coculture was propagated for 4 and 7 days to facilitate NMJ formation, for a total of 9 and 12 days of tissue propagation from the thawing of myoblasts (Fig. 3A).

Fig. 3B shows the cell morphology of the day 9 NMJ coculture. The dotted area represented the myotubes, while the arrowheads indicated the nerve fibers, demonstrating that the MNs projected nerve fibers into the SKM layers. In Fig. 3C, a low-magnification ICC image revealed an extensive network of NF/SV2^+^ neurite projections with significant overlap with TTN, along with the expression of numerous AChR clusters, which are key receptors for receiving neurotransmitters from MNs, demonstrating the integration of MNs and SKMs. Furthermore, high AChR expression was observed on TTN^+^ myotubes, with significant overlap with NF/SV2-labeled neurons (represented by arrowheads in Fig. 3D). Confocal imaging confirmed the colocalization of TTN, NF/SV2, and AChR at the NMJ endplate-like sites (represented by arrowheads in Fig. 3E), indicating the specificity of NMJ formation. Spontaneous myotube contractions were observed in the day 9 NMJ model (Videos S1). On day 9, contacts were observed between NF/SV2, TTN and AChR. By day 12, high colocalization of NF/SV2 and AChR was observed on the endplate-like structures (Fig. 3F), indicating the maturation of NMJs. To assess their functional maturation, NMJs were stimulated with CaCl_2_, as described previously [25, 26]. Continuous changes in intensity were observed in regions of interest (ROIs) in NMJs on day 9 after CaCl_2_ treatment; however, these changes lacked a clear rhythmic pattern, indicating no significant rhythmic SKM contractions following the treatment (Fig. 3G). This result demonstrated the progression of NMJ formation, but maturation had not yet occurred by day 9. By day 12, 2–25 mM CaCl_2_ successfully elicited significant rhythmic SKM contractions in NMJs, but not in the SKMs alone. Furthermore, both SKM contractions on day 9 and day 12 NMJs were inhibited with an AChR antagonist (NMJ inhibitor) (Fig. 3H), indicating that the contractions were innervated by MNs. These findings confirmed the successful establishment of functional NMJs within 12 days after thawing the SKMs.

### No significant differences were observed between the properties of SOD1^G85R^ ALS and SOD1^G85G^ isogenic healthy SKMs

To evaluate the applicability of the NMJ model for ALS, we prepared stocks of SKM and MN cells from SOD1^G85R^ mutant ALS and SOD1^G85G^ CRISPR/Cas9-corrected, healthy isogenic iPSC lines. These two iPSC lines had been previously established and characterized and had generated high-purity MN cryopreservation stocks [33, 34]. The SOD1^G85R^ MNs exhibited several ALS-specific cytopathies, including nerve fiber beads, neurite degeneration, and neurotransmitter hypersensitivity, as identified previously [34].

**Fig. 4:**
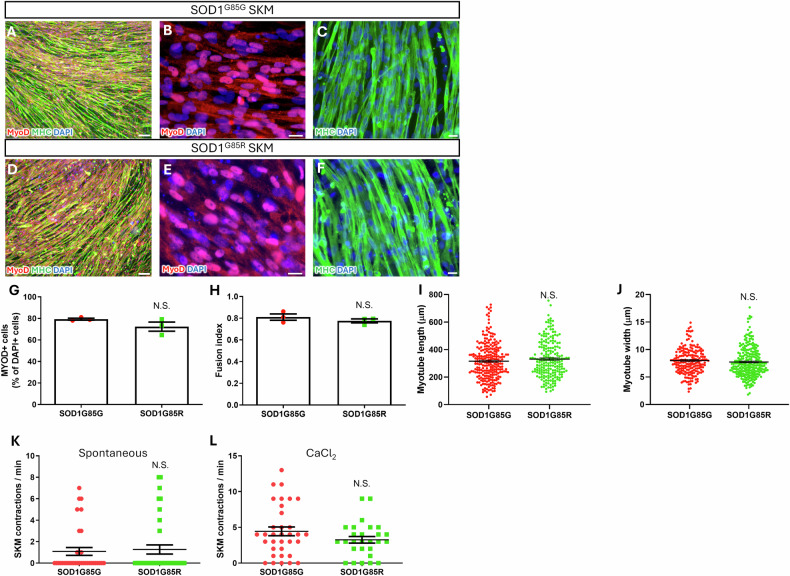
Generation and identification of day 12 maturated skeletal muscles (SKM) from SOD1^G85G^ and SOD1^G85R^ induced pluripotent stem cell (iPSC) lines. **A**–**F** Immunocytochemistry (ICC) staining for SKM marker MYOD and MHC on SOD1^G85G^ (**A**–**C**) and SOD1^G85R^ (**D**–**F**) SKMs. Calculations of MYOD expression (**G**), myotube fusion index (**H**), myotube length (**I**), and width (**J**) of SOD1^G85G^ and SOD1^G85R^ SKMs. Spontaneous (**K**) and CaCl_2_-induced (**L**) SKM contraction ratios in SKMs. **G**, **H** For each group, 6 images were analyzed from 3 independent experimental replicates, with each data point representing the mean value of a single replicate. **I** In total, 250 (SOD1^G85G^) and 206 (SOD1^G85R^) myotubes from 6 images were analyzed, with each dot representing one myotube. **J** In total, 198 (SOD1^G85G^) and 252 (SOD1^G85R^) myotubes from 6 images were analyzed, with each dot representing one myotube. **K** In total, 34 and 37 ROIs were analyzed, with each dot representing one ROI. **L** A total of 34 and 27 ROIs were analyzed, with each dot representing one ROI. All data were collected from 3 independent experimental replicates. For (**G**–**L**), the unpaired 2-tailed Student’s *t*-test was used. Scale bars: 100 μm (**A**, **D**); and 20 μm (**B**, **C**, **E**, **F**).

In this study, we further investigated the presence of any potential ALS-specific cytopathies in the *SOD1*^G85R^ SKMs. ICC staining performed after differentiation of the two iPSC lines into SKMs demonstrated the expression of SKM-specific markers MYOD and MHC in SOD1^G85G^ and SOD1^G85R^ SKMs (Fig. 4A–F). To evaluate potential ALS cytopathies in SKMs, we evaluated MYOD expression, myotube fusion index, and myotube length and width in SOD1^G85G^ and SOD1^G85R^ SKMs. No significant differences were observed in any of these measured indices (Fig. 4G–J). To evaluate the functional differences between SOD1^G85G^ and SOD1^G85R^ SKMs, the rates of unstimulated spontaneous contractions and CaCl_2_-stimulated contractions were recorded. The results indicated no significant differences in either spontaneous or CaCl_2_-stimulated contractions between SOD1^G85G^ and SOD1^G85R^ SKMs (Fig. 4K, L). Therefore, no ALS cytopathies were observed in SOD1^G85R^ SKMs.

### SOD1^G85R^ MNs, but not SKMs, decreased AChR expression in the 9-day cross-coculture NMJ model

The primary initiator of NMJ cytopathies in ALS remains unclear. Our NMJ model, which is capable of cross-coculturing healthy and diseased MNs and SKMs, could potentially elucidate the initial mechanisms and the respective roles of MNs and SKMs in ALS.

**Fig. 5:**
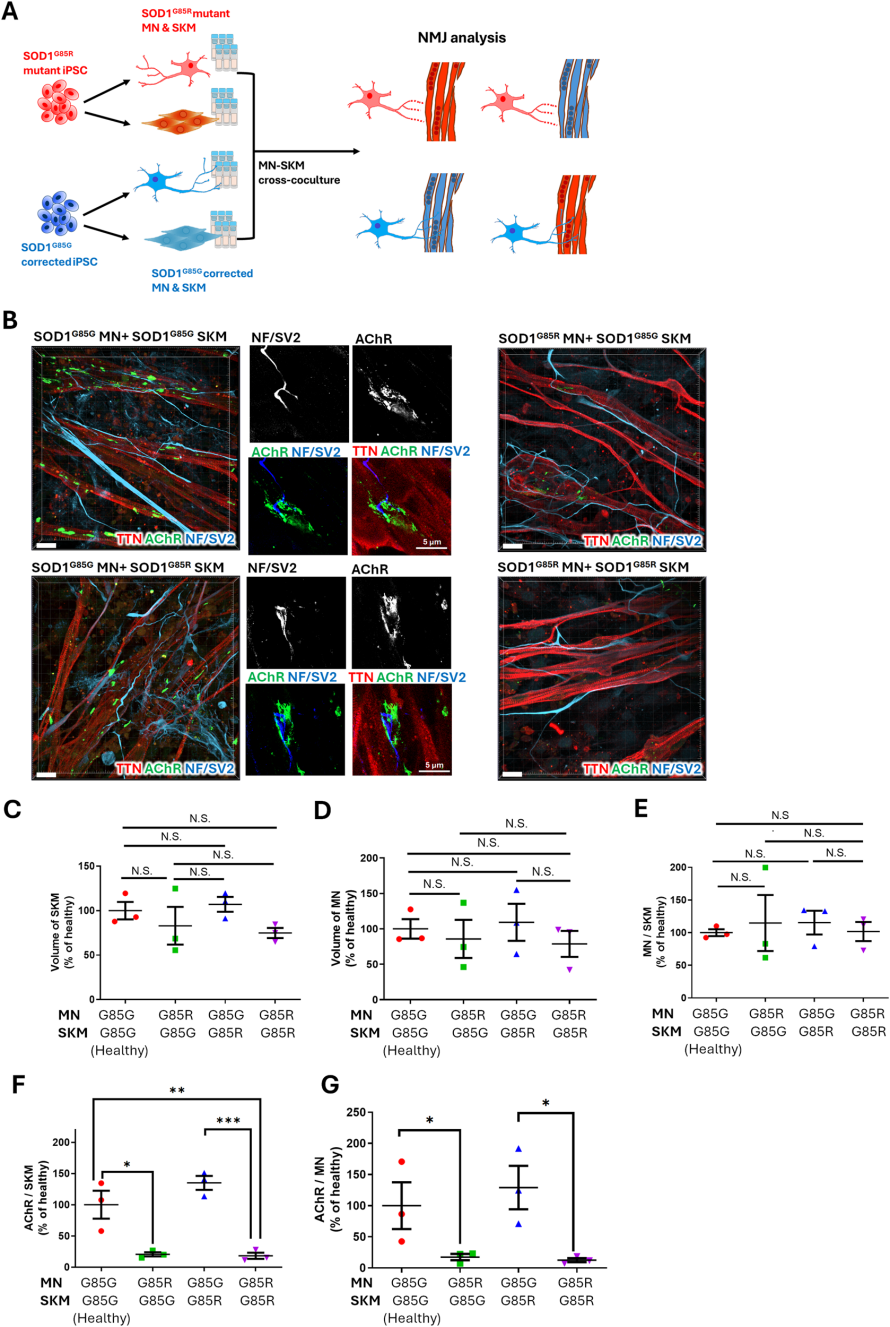
Volume of skeletal muscles (SKMs), motor neurons (MNs), and acetylcholine receptors (AChRs) in the neuromuscular junction (NMJ) model derived from the cross-coculture of SOD1^G85G^ and SOD1^G85R^ MNs and SKMs. **A** Diagram of the cross-coculture of SOD1^G85R^ and SOD1^G85G^ MNs and SKMs. **B** 3D confocal images of immunocytochemistry (ICC) staining for the SKM marker TTN; neuronal marker NF; and NMJ markers SV2 and AChR. **C**–**G** Calculations of the volume of SKMs (**C**), MNs (**D**), and the ratios of MN/SKM (**E**), AChR/SKM (**F**), and AChR/MN (**G**) in day 9 cross-coculture NMJ models. Five (SOD1^G85G^ MN + SOD1^G85G^ SKM), 4 (SOD1^G85R^ MN + SOD1^G85G^ SKM), 8 (SOD1^G85G^ MN + SOD1^G85R^ SKM) and 6 (SOD1^G85R^ MN + SOD1^G85R^ SKM) images from 3 independent experimental replicates were analyzed, with each data point representing the mean value from a single replicate. The one-way analysis of variance with Tukey’s post hoc test was used. The schematic in (**A**) was generated using Microsoft PowerPoint. Scale bar: 20 μm in confocal 3D images; and 5 μm in 2D image slices.

The procedure for the cross-coculture NMJ model is illustrated in Fig. 5A. After 5 days of SKM maturation and 4 days of MN–SKM coculture (9 days from myoblast thawing), the NMJ model was subjected to ICC. The 3D confocal images of each group were shown in Fig. 5B and Videos S2–S5: (1) SOD1^G85G^ MN + SOD1^G85G^ SKM (as healthy control, Video S2); (2) SOD1^G85R^ MN + SOD1^G85G^ SKM (Video S3); (3) SOD1^G85G^ MN + SOD1^G85R^ SKM (Video S4); and (4) SOD1^G85R^ MN + SOD1^G85R^ SKM (Video S5). The images in Fig. 5B revealed the expression of AChR and the formation of NMJ endplate-like structures in the NMJs of groups (1) and (3) (derived from SOD1^G85G^ MN cocultures). In contrast, the NMJs of groups (2) and (4) (derived from SOD1^G85R^ MN cocultures) showed a disappearance of AChR.

To quantify the potential NMJ cytopathies in ALS, we measured the volumes of SKMs and MNs and calculated the MN/SKM, AChR/SKM, and AChR/MN ratios for each NMJ group (Fig. 5C–G). No significant differences were observed in the volumes of SKMs (Fig. 5C), MNs (Fig. 5D), and MN/SKM ratios (Fig. 5E) in all groups. However, AChR expression significantly decreased in the groups cocultured with SOD1^G85R^ MNs. Specifically, SOD1^G85R^ MNs reduced the AChR/SKM ratio to 20.65% ± 3.3% (SOD1^G85R^ MN + SOD1^G85G^ SKM) and 18.25% ± 5.02% (SOD1^G85R^ MN + SOD1^G85R^ SKM) compared to the healthy control (Fig. 5F). Similar reductions were noted in the AChR/MN ratio, with SOD1^G85R^ MNs decreasing the ratio to 17.49% ± 5.13% (SOD1^G85R^ MN + SOD1^G85G^ SKM) and 12.55% ± 3.26% (SOD1^G85R^ MN + SOD1^G85R^ SKM) compared to the healthy control (Fig. 5G). Significant decreases in the AChR/SKM and AChR/MN ratios involving SOD1^G85R^ MNs were also observed within the paired groups of SOD1^G85G^ MN + SOD1^G85R^ SKM and SOD1^G85R^ MN + SOD1^G85R^ SKM, both cocultured with the same SOD1^G85R^ SKMs. (Fig. 5F and G). These results indicated that SOD1^G85R^ ALS MNs directly influence AChR expression.

In contrast, SOD1^G85G^ and SOD1^G85R^ SKMs did not significantly affect AChR expression, as shown by the AChR/SKM and AChR/MN ratios within the paired groups of SOD1^G85G^ MN + SOD1^G85G^ SKM and SOD1^G85G^ MN + SOD1^G85R^ SKM, as well as SOD1^G85R^ MN + SOD1^G85G^ SKM and SOD1^G85R^ MN + SOD1^G85R^ SKM (Fig. 5F, G). These results indicated that MNs are the major initiators of AChR cytopathies in the SOD1^G85R^ mutant ALS NMJ model.

### SOD1^G85R^ MNs altered the properties of AChR and reduced calcium-induced SKM contractions in the 12-day NMJ model

**Fig. 6:**
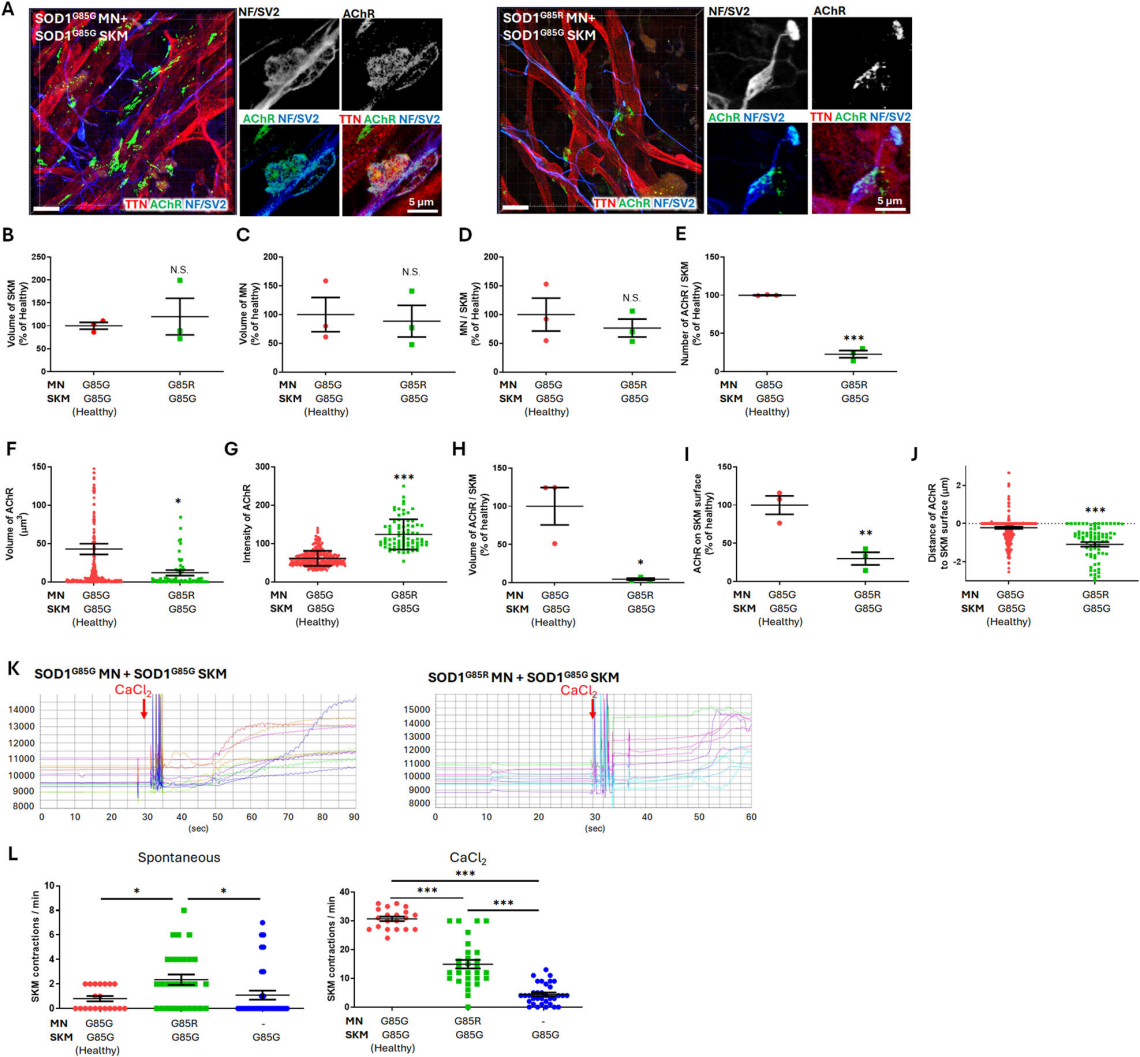
Functions and properties of the neuromuscular junction (NMJ) derived from the coculture of SOD1^G85G^ and SOD1^G85R^ motor neurons (MNs) with SOD1^G85G^ skeletal muscles (SKM). **A** 3D confocal images of immunocytochemistry (ICC) staining for the SKM marker TTN; neuronal marker NF; and NMJ markers SV2 and AChR. **B**–**J** Calculations of the volume of SKM (**B**), MN (**C**), and MN/SKM ratio (**D**). Analysis of AChR properties, including number (**E**), volume (**F**), intensity (**G**), and AChR/SKM ratio (**H**) in coculture NMJ models. Distribution analysis of AChRs depicting the ratio of AChR on the SKM surface (**I**) and distances from the SKM surface (**J**). **K** Bright-field intensity changes in different ROIs after CaCl_2_ induction in coculture NMJs. **L** Spontaneous and CaCl_2_ induced SKM contraction ratios in NMJs. **B**–**E**, **H**, **I** For each group, 6 images were analyzed from 3 independent experimental replicates, with each data point representing the mean value of a single replicate. **F**, **G**, **J** A total of 333 (SOD1^G85G^ MN + SOD1^G85G^ SKM) and 74 (SOD1^G85R^ MN + SOD1^G85G^ SKM) AChRs from 6 images were analyzed, with each dot representing one AChR. **L** In total, 20 (SOD1^G85G^ MN + SOD1^G85G^ SKM), 29 (SOD1^G85R^ MN + SOD1^G85G^ SKM) and 34 (SOD1^G85G^ SKM) ROIs were analyzed, with each dot representing one ROI. All data were collected from 3 independent experimental replicates. The unpaired 2-tailed Student’s *t*-test was used in (**B**–**J**) and one-way analysis of variance with Tukey’s post hoc test was used in (**L**). “−” indicating inside, “0” indicating directly on the surface, and “+” indicating outside the SKM surface in (**J**). Scale bar: 20 μm in confocal 3D images; and 5 μm in 2D image slices.

To further investigate the impact of SOD1 mutant MNs on ALS NMJ cytopathies, we cocultured SOD1^G85G^ and SOD1^G85R^ MNs with healthy SOD1^G85G^ SKMs for 7 days (totaling 12 days following myoblast thawing) to establish mature NMJs capable of responding to calcium-induced myotube contractions. Figure 6A and Videos S6 and S7 show 3D confocal images of the following NMJ cocultures: SOD1^G85G^ (healthy control, Video S6) and SOD1^G85R^ MNs (Video S7) cocultured with healthy SOD1^G85G^ SKM. Images in Fig. 6A showed endplate-like structures in both NMJs. The healthy control exhibited large and well-structured NMJs, whereas the SOD1^G85R^ cocultured NMJs displayed small and fragmented structures. No significant differences were observed in the volumes SKMs and MNs, or in the MN/SKM ratio (Fig. 6B–D), consistent with the 9-day NMJ model shown in Fig. 5C–E. Thus, SOD1 mutant MNs did not affect the fundamental properties of MNs and SKMs in the 12-day NMJ model. However, we observed a reduction in the number of AChRs (22.82% ± 4.69%) in NMJs with SOD1^G85R^ MNs compared to those with SOD1^G85G^ MNs (Fig. 6E). Additionally, the volume of individual AChRs decreased from 42.93 ± 7.03 μm³ to 12.03 ± 3.59 μm³ in NMJs with SOD1^G85R^ MNs (Fig. 6F). Conversely, the intensity of AChRs increased from 61.8 ± 19.66 to 124.1 ± 39.51 in NMJs with SOD1^G85R^ MNs (Fig. 6G). The total AChR volume in the SOD1^G85R^ MN group decreased to 4.53% ± 2.35% compared to the SOD1^G85G^ MN group (Fig. 6H). Analysis of AChR distribution revealed a significant decrease in AChR expression on the surface of SKMs in NMJs with SOD1^G85R^ MNs, accounting for only 29.97% ± 14.34% of the healthy control (Fig. 6I). The AChR distribution in NMJs with SOD1^G85R^ MNs was predominantly located inside the SKM rather than on its surface (Fig. 6J). Thus, SOD1 mutant MNs not only decrease AChR expression but also alter their distribution and properties, providing insights into the mechanisms underlying ALS NMJ cytopathies.

To assess the functional impact of SOD1^G85R^ MNs on NMJs, NMJs were stimulated with CaCl_2_ treatment. Bright-field videos before and after CaCl_2_ treatment were shown in Videos S8 (NMJ with SOD1^G85G^ MN) and S9 (NMJ with SOD1^G85R^ MN). Intensity changes can be observed in ROIs (Fig. 6K). NMJs with SOD1^G85G^ MNs exhibited rhythmic SKM contractions after CaCl_2_ addition, whereas NMJs with SOD1^G85R^ MNs showed delayed and unclear SKM contractions. The rates of unstimulated spontaneous contractions and CaCl_2_-stimulated contractions in NMJs with SOD1^G85G^ and SOD1^G85R^ MNs, as well as those in SKMs alone, were recorded (Fig. 6L). NMJs with SOD1^G85R^ MNs exhibited higher rates of spontaneous contractions than both NMJs with SOD1^G85G^ MNs and SKM alone. After CaCl_2_ stimulation, the average SKM contraction rate in SOD1^G85R^ NMJs decreased to 14.93 ± 1.5 contractions/min, which was lower than the 30.7 ± 0.79 contractions/min observed in SOD1^G85G^ NMJs. SKM alone exhibited the lowest contraction rate, averaging 4.44 ± 0.61 contractions/min, reflecting the baseline contraction rate in the absence of MN innervation. SOD1 mutant MNs significantly influenced ALS cytopathies, affecting the number, volume, and distribution of AChR, as well as the spontaneous and induced SKM contractions innervated by MNs.

### SOD1^D90A^ MNs decreased AChR expression and calcium-induced SKM contractions in NMJ

**Fig. 7:**
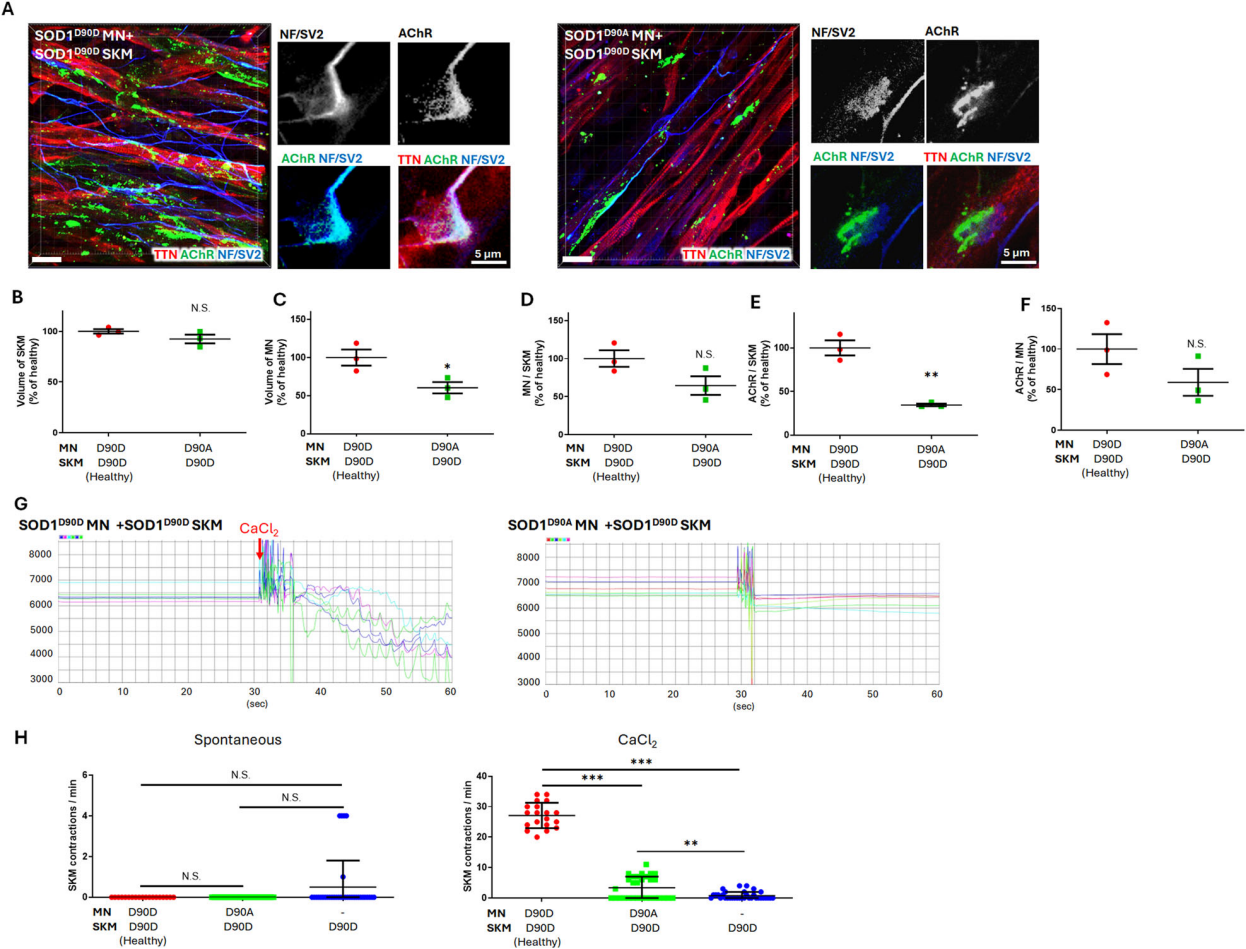
Functions and properties of the neuromuscular junction (NMJ) model derived from the coculture of SOD1^D90D^ and SOD1^D90A^ motor neurons (MNs) and SOD1^D90D^ skeletal muscles (SKM). **A** 3D confocal images of immunocytochemistry (ICC) staining for the SKM marker TTN; neuronal marker NF; and NMJ markers SV2 and AChR. **B**–**F** Calculations of the volume of SKM (**B**), MN (**C**), and the ratios of MN/SKM (**D**), AChR/SKM (**E**), and AChR/MN (**F**) in all cross-coculture NMJ models. For each group, 5 images were analyzed from 3 independent experimental replicates, with each data point representing the mean value of a single replicate. **G** Bright-field intensity changes in different ROIs after CaCl_2_ induction in coculture NMJs. **H** Spontaneous and CaCl_2_-induced SKM contraction ratios in NMJs. In total, 19 (SOD1^D90D^ MN + SOD1^D90D^ SKM), 30 (SOD1^D90A^ MN + SOD1^D90D^ SKM), and 34 (SOD1^D90D^ SKM) ROIs were analyzed, with each dot representing one ROI. All data were collected from three independent experimental replicates. The unpaired 2-tailed Student’s *t*-test was used in (**B**–**F**) and one-way analysis of variance with Tukey’s post hoc test was used in (**H**). Scale bars: 20 μm in confocal 3D images; and 5 μm in 2D image slices.

To validate the reproducibility of our rapid NMJ model and confirm the findings on SOD1^G85R/G85G^ iPSCs, we established NMJ models using another pair of SOD1 mutant iPSC lines: SOD1^D90A^ ALS and SOD1^D90D^ healthy isogenic iPSC lines. The NMJ formation followed the same procedure as described above. Confocal microscopy was used to capture 3D ICC images of NMJ cocultures: SOD1^D90D^ (healthy NMJ, Video S10) and SOD1^D90A^ MNs (Video S11) with SOD1^D90D^ healthy SKM (Fig. 7A), which were subsequently analyzed (Fig. 7B–F). Images in Fig. 7A showed endplate-like structures in both NMJs.

In NMJs with SOD1^D90A^ MNs, the MN volume significantly decreased to 60.44% ± 7.41%, while the MN-to-SKM ratio exhibited a decreasing trend to 64.44% ± 12.29%, though this reduction was not statistically significant (Fig. 7C, D). These findings demonstrated the potential degeneration of SOD1^D90A^ MNs. Furthermore, the AChR/SKM ratio decreased to 34.62% ± 1.5% compared to healthy control (Fig. 7E), indicating a reduction in AChR expression in NMJs with SOD1^D90A^ MNs. However, although the AChR/MN ratio showed a decreasing trend to 58.97% ± 16.57%, this change was not significant (Fig. 7F) and may be attributed to a reduction in MN volume at the NMJs with SOD1^D90A^ MNs.

The NMJ functional test revealed repeating contractions in SOD1^D90D^ MN NMJs (Video S12) and almost no response in SOD1^D90A^ MN NMJs (Video S13) to CaCl_2_ treatment. The intensity changes in ROIs are shown in Fig. 7G. Calculations demonstrated no significant differences in the spontaneous contraction rates between SOD1^D90D^ and SOD1^D90A^ MN-based NMJs, as well as SKM alone (Fig. 7H). However, after CaCl_2_ stimulation, the contraction rate significantly decreased in SOD1^D90A^ MN-based NMJs compared to SOD1^D90D^, while SKM alone exhibited the lowest contraction rate (Fig. 7H). These results demonstrated the SOD1^D90A^ MNs significantly reduced AChR expression and impaired SKM contractions innervated by MNs at the NMJ.

## Discussion

In the cross-coculture experiment, we observed significant changes in the number, individual volume, and intensity of AChR in NMJs with SOD1^G85R^ ALS MNs (Fig. 6). These changes have not been previously observed in any human NMJ models. Notably, muscle biopsies from patients with early-stage ALS have shown that vacant and reinnervated NMJs exhibited fragmented, small-volume, and high-intensity AChR expression [38]. These findings mirror those observed in our NMJ model, underscoring its relevance to actual patient tissues.

Figures 6I, J showed the changes in AChR distribution in NMJs with SOD1^G85R^ MNs, with AChRs primarily located within the myotubes rather than on the surface. We hypothesized that NMJ degeneration disrupts AChR recycling and homeostasis, leading to AChR endocytosis in dysfunctional NMJs. While some studies have evaluated the impairment of AChR trafficking and recycling in aged and ALS NMJs [39, 40], the underlying mechanisms remain largely unclear. Further analysis of AChR trafficking in ALS NMJs must be conducted to elucidate these mechanisms in the future.

Although G85R and D90A SOD1 mutant NMJs exhibited AChR and functional cytopathies, the severity of these cytopathies differed between the two mutants. Compared with the G85R variants, D90A SOD1 NMJs showed more severe neurite degeneration and almost no contraction response to CaCl_2_ stimulation (Fig. 7). Our previous report also demonstrated that SOD1^D90A^ MNs exhibit more severe nerve fiber degeneration and glutamate hypersensitivity than SOD1^G85R^ MNs [34]. Overall, these results indicate that the D90A SOD1 mutant causes more severe ALS cytopathies in the MNs that may directly cause NMJ defects. These findings emphasize the need to identify the specific SOD1 mutation when studying ALS pathology and developing targeted therapies. The distinct differences in cytopathies between G85R and D90A mutants highlight the complexity of ALS and the necessity of tailored approaches in modeling and treatment.

Similar to our findings, a previous report found that the D90A SOD1 mutant caused MN neurite degeneration [19]. Additionally, it was highlighted that both SOD1 mutant MNs and SKMs derived from iPSCs exhibited ALS cytopathies, including changes in the number, fidelity, and stability of NMJ functions [22]. In our study, we demonstrated that SOD1 mutant MNs predominantly altered the NMJ structure and properties of AChRs. These findings provide a comprehensive perspective from various analytical angles, aiding in the discovery of detailed insights into ALS. Interestingly, another study discovered that the E100G and L144P SOD1 mutations led to significant SKM defects, such as decreases in width, length, thickness, and fusion index [41]. However, no significant changes in these parameters were observed in the G85R and D90A SOD1 mutant SKMs (Fig. 4). Despite differences in experimental design and analytical approaches among the different studies conducted, the type of SOD1 mutation may be the primary cause of these differences. Thus, personalized disease models that precisely mimic the disease manifestations are required to understand such diseases in individual patients.

The first observable pathological defect in patients with ALS occurs in the NMJ [10–12], even before observable MN degeneration. However, the major mechanisms and causes of NMJ cytopathies in ALS remain largely unclear. Several “neurocentric” hypotheses have been proposed, highlighting the dominant role of MNs [42]. Other cell types at the NMJ, including SKMs and glial cells, have recently been reported as potential key players in triggering degeneration [42]. The “dying back” hypothesis suggests that retrograde signals, such as Nogo-A secreted by muscles, can disrupt the NMJ structure, inhibit MN neurite elongation, or cause MN degeneration [41–44]. Several ALS clinical trials are specifically investigating muscles [45–48], highlighting the potential key role of muscles in ALS progression. However, the roles of MNs and muscles in ALS NMJ progression are still not fully explored because of the lack of patient-derived NMJ models that enable healthy–pathogenic cell cross-coculture. Very few research groups have conducted patient-derived NMJ models to identify the potential roles of MNs and SKMs in ALS [22]. In this study, we developed a human NMJ model that enabled a healthy–pathogenic cell cross-culture to investigate early NMJ dysfunction in ALS. Although our results demonstrate only preliminary hints and are restricted to ALS with specific SOD1 mutations, they provide a workable platform for uncovering the mechanisms of NMJ defects in ALS. This model can potentially advance our understanding of NMJ pathophysiology in ALS, as well as those of other neuromuscular diseases.

Previous iPSC-based NMJ models have primarily relied on MN–SKM coculture and self-organized 3D neuromuscular organoids [17–32]. Given the technological challenges of mature neuron cryopreservation, MN–SKM coculture-based NMJs typically begin with MN differentiation, requiring a continuous, uninterrupted period of at least 24–47 days to form functional NMJs [17–24, 27, 28, 32]. This process, which must be synchronized with the timeline of muscle cell differentiation, is complex and time-consuming. Self-organized 3D neuromuscular organoids establish a precise NMJ model that mirrors the developmental processes of human NMJs, thereby facilitating NMJ formation research. However, even with MYOD overexpression, the development of functional NMJs takes months [25, 26, 29–31]. Technical challenges and quality control issues further limit the large-scale application of self-organized 3D neuromuscular organoids, thereby highlighting the need for more efficient and scalable NMJ modeling approaches. In this study, we established a rapid NMJ model derived entirely from human cells using iPSCs that were individually differentiated into MNs and SKMs and then cryopreserved. The quality and purity of each cell type were carefully controlled during preparation. Producing an NMJ model with proper structures, specific marker expression, and inducible myotube contractions from cryopreserved stocks took only 12 days. The entire process of establishing the model is straightforward and easy to manage. Thus, our model addresses these challenges by providing a quicker, more streamlined method for establishing NMJs, potentially advancing NMJ research and therapeutic development.

In addition to developing a rapidly established human NMJ model, our study utilized 3D imaging to analyze these models, revealing potential novel ALS cytopathies in the number, volume, intensity, and distribution of AChRs. This research not only successfully recapitulated ALS-specific cytopathies observed in human tissues but also identified novel potential ALS cytopathies in NMJs. Thus, this study highlights the value of our NMJ model in advancing the understanding of NMJ pathophysiology and its potential utility in developing therapeutic interventions for ALS.

**Fig. 8:**
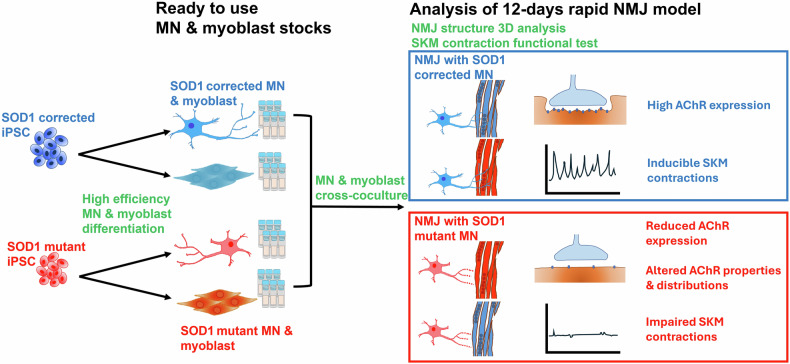
Schematic illustrating the rapid human neuromuscular junction (NMJ) model and its application to amyotrophic lateral sclerosis (ALS) modeling. We developed human NMJ-like tissues within 12 days using cryopreserved motor neurons (MNs) and iPSC-derived skeletal myoblasts and employed this model in ALS studies. ALS-specific cytopathies similar to those observed in patient tissues were observed, and the critical role of MNs in initiating NMJ cytopathies in ALS was evaluated. This figure was generated using Microsoft PowerPoint.

The conclusion of this study was summarized as Fig. 8. Here we developed a rapidly established, user-friendly NMJ model from iPSCs, capable for mechanistic studies and drug testing in motor function disease modeling. This model represents a significant advancement in NMJ disease research, providing a practical tool for understanding disease mechanisms and evaluating novel therapeutic interventions.

## Materials and methods

### Pluripotent stem cell culture and expansion

The SOD1^D90A^ and SOD1^D90D^ iPSC lines were purchased from WiCell (Madison, WI, USA) [35]. The SOD1^G85R^ ALS iPSC line was generated from PBMCs obtained from patients according to the guidelines of the Declaration of Helsinki and with the approval of the Ethical Institutional Review Board of Hualien Tzu Chi Hospital (IRB105-131-A). SOD1^G85G^ iPSCs were generated from SOD1^G85R^ iPSCs by CRISPR/Cas9 point correction [34]. Informed consent was obtained from the patient. The iPSC properties were previously identified. Briefly, PBMCs were cultured in StemPro-34 SFM (ThermoFisher Scientific, Waltham, MA, USA) and supplemented with SCF, FLT-3, IL-3, and IL-6 (Peprotech, Cranbury, NJ, USA). Reprogramming was performed using CytoTune iPS 2.0 Sendai Reprogramming Kit (ThermoFisher Scientific) following the manufacturer’s instructions [49]. All iPSC lines were transferred onto 1% Geltrex (ThermoFisher Scientific)-coated culture dishes and expanded in TeSR-E8 medium (StemCell Technologies, Vancouver, BC, Canada) or Pluto human iPS/ES cell culture medium (DuoGenic Stem Cells, Taichung, Taiwan). The iPSCs could also be successfully cultured and expanded in Duo-ESy iPS/ES culture medium [50] (DuoGenic Stem Cells) without requiring a pre-coating process. iPSCs were passaged after 3–5 days using Accutase (Merck-Millipore, Billerica, MA, USA) and then reseeded at 1:5 to 1:10 ratios. All culture media were refreshed daily. The cells were authenticated through karyotyping and SOD1 mutant point sequencing. Mycoplasma testing was performed every 10 passages using the MycoSEQ™ Plus Mycoplasma Detection Kit (ThermoFisher Scientific).

### MNP differentiation, MN maturation, cryopreservation, and thawing

The CHSF–MN differentiation protocol was performed, as described previously [33]. Briefly, when the iPSCs reached 80% confluence, the cells were treated with 1 mg/mL Dispase II (Sigma-Aldrich, St. Louis, MO, USA) for 5–15 min until the periphery of the colonies started to round up. The cells were washed twice with DMEM-F12 and then lifted by scraping. The clumps were dissociated into ~200-μm clusters by pipetting with a P1000 pipetman and transferred into ultralow attachment dishes in TeSR-E6 medium (StemCell Technologies) for 24 h to form serum-free culture of embryoid bodies (EBs). For successful EB formation, 5 μM ROCKi Y27632 (Cayman Chemical, Ann Arbor, MI, USA) was added during the first 4 h to avoid PSC blebbing. On day 1, the EBs were filtered through 400 μm cell strainers (pluriSelect, El Cajon, CA, USA) to remove large aggregates before undergoing CHSF–MN neural induction. The neural induction medium consisted of DMEM-F12 with 1% N2 supplement, 1 mM nonessential amino acids (NEAAs), and 2 mM glutamate (all from ThermoFisher Scientific). The neural induction factors for CHSF, namely, 3 μM CHIR99021 (Cayman Chemical), 10 μM SB431542 (Sigma-Aldrich), and 10 ng/mL recombinant human FGF-2 (R&D Systems, Minneapolis, MN, USA), were added on days 1 to 4 without changing the medium. On day 4, the neural induction media were changed to neurobasal media (ThermoFisher Scientific) containing 1% N2 supplement, 1 mM NEAAs, 2 mM glutamate, and MNP patterning factors (3 μM CHIR99021, 2 μM SB431542, and 200 nM LDN193189; Cayman Chemical), 0.5 μM SAG (Cayman Chemical), and 0.5 μM RA (Sigma-Aldrich) until day 25 of differentiation. On day 15, 2% B27 supplement (ThermoFisher Scientific) was added. After day 25, morphogens were withdrawn from the culture medium. The EBs were mechanically pipetted at 200- to 300-µm clusters every 2–3 days with a P1000 pipetman and cultured for at least another 4–8 weeks. After the end of the neural induction step on day 4, the culture media were changed every other day.

MN cryopreservation, thawing, and maturation were performed, as described previously [33]. On day 28, EBs were dissociated into 200- to 300-µm clumps by mechanical pipetting with a P1000 pipetman. The number of EBs in 500 μl suspension was counted, and the total number of EBs was calculated, followed by centrifugation at 1000 rpm for 2 min. We removed the supernatant, gently tapping the tube to dissociate the pellet. We suspended ~100 EBs in 1 mL STEM-CELLBANKER Stem Cell Freezing Media (Amsbio) supplemented with ROCKi and 1% RevitaCell (ThermoFisher Scientific) and transferred them to 1-mL cryotubes. The cryotubes were placed in a Cryo 1 °C freezing container (Nelgene) precooled to 4 °C and filled with fresh isopropanol. The freezing container was stored at −80 °C in a refrigerator for 24 h and transferred to a liquid nitrogen tank for long-term storage. For thawing, the cryotube was thawed in a 37 °C water bath until only a small amount of unmelted ice remained. EBs were quickly transferred into a 15-mL centrifuge tube, immediately supplemented with 2 mL DMEM-F12, and centrifuged at 1000 rpm for 2 min. The supernatant was discarded, the tube was tapped to loosen the pellet, and the EBs were transferred to an ultralow culture dish containing neurobasal medium supplemented with 1% N2, 1 mM NEAAs, 2 mM glutamate, and 2% B27. In the first 24 h of thawing, 1% RevitaCell supplement was also added. For adherent culture or further experiments, EBs were dissociated using Accutase for 5 min, with gentle mechanical pipetting every minute with a P1000 pipetman to create cell clumps smaller than 100 μm, and centrifuged at 1000 rpm for 2 min, then seeded onto 1% Geltrex-coated cell culture dishes supplemented with 0.2 μM compound E (Cayman Chemical), brain-derived neurotrophic factor (Peprotech), glial cell line-derived neurotrophic factor (Peprotech), and dbcAMP (Sigma-Aldrich) for 3 days. During the first 4 h of cell adhesion, 1% RevitaCell supplement (ThermoFisher Scientific) was added to the cells. To generate non-motor neurons, SAG was replaced with 10 μg/ml cyclopamine, a sonic hedgehog inhibitor, during the differentiation process.

### Skeletal myoblast differentiation, expansion, cryopreservation, and SKM maturation

Skeletal myoblast differentiation: On day −1, iPSCs were dissociated into single cells with Accutase and seeded on Geltrex-coated culture plates at 35,000 cells/cm^2^ in the TeSR-E6 medium with 5 μM Y27632. The next day, day 0, the medium was switched to basal differentiation medium composed of DMEM-F12 with 1% Gibco Insulin-Transferrin-Selenium. From days 0 to 2, 3 μM CHIR99021 was added to the culture. From day 2 to day 4, cells were switched to basal differentiation medium supplemented with 0.2 μM LDN193189 and 5 μM SB431542. On days 4–6, culture medium was changed to basal differentiation medium supplemented with 3 μM CHIR and 20 ng/mL FGF2. On day 6, the culture medium was changed to DMEM with 15% Knockout Serum Replacement and supplemented with 10 ng/mL HGF and 2 ng/mL IGF1 for 22 days.

Skeletal myoblast expansion and cryopreservation: on day 28, cells were washed with PBS and detached with Accutase. The cell suspension was filtered sequentially through 50-mm cell strainers to exclude cell aggregates and generate single-cell suspensions. Filtered cells were resuspended in MyoCult™-SF Expansion Medium with 10 μM Y27632 and plated at a density of 20,000 cells/cm^2^ onto Geltrex-coated tissue culture plates. The next day, the culture medium was changed to MyoCult™-SF Expansion Medium without Y27632 for expansion. The cells were passaged using Accutase and expanded every 2 days for a total of five passages (10 days of expansion). They were then cryopreserved using STEM-CELLBANKER Stem Cell Freezing Medium (Amsbio). All experiments in this manuscript were conducted using cryopreserved myoblast stocks from passage 5.

SKM maturation: Thawed myoblasts were seeded at a density of 120,000–150,000 cells/cm² in MyoCult™ Differentiation Medium on Geltrex-coated plates, ensuring over 90% confluence after cell adhesion. The cultures were incubated at 37 °C with 5% CO_2_ for 5 days. On day 5, half of the medium was replaced with fresh MyoCult™ Differentiation Medium, and incubation continued for up to 7 more days. By day 12, the cells were ready for downstream applications.

### In vitro NMJ formation

A 15-mm round coverslip was placed in each well of a 24-well plate. Myogenic progenitor cells were seeded into the Geltrex-coated wells at a density of 250,000–300,000 cells per well (120,000–150,000 cells/cm^2^). MyoCult™ Differentiation Medium was added at 750 μL to each well, and the cultures were incubated for 5 days. Multinucleated myotubes gradually formed and their proportion increased over this period. On day 5, the differentiation medium was replaced with neurobasal medium (ThermoFisher Scientific) supplemented with 1% N2, 1 mM NEAAs, 2 mM glutamate, 2% B27, and 1% RevitaCell Supplement (ThermoFisher Scientific). Subsequently, MN spheres were thawed and suspended in a total of 5 mL of NB medium. The number of spheres in 1 mL cell suspension was counted, followed by centrifugation at 1000 rpm for 2 min. Fifty spheres were then dissociated using Accutase for 5 min, with gentle mechanical pipetting every minute with a P1000 pipetman to create cell clumps smaller than 100 μm, seeded onto the differentiated myotubes. The day after seeding the MN EBs, RevitaCell Supplement was replaced with 0.2 μM compound E (Cayman Chemical). The medium was changed every 2 days. The cells were cocultured for 4–7 days, after which the NMJs could be analyzed.

### ICC

The cells were seeded in 24-well plates, fixed with 4% paraformaldehyde (Sigma-Aldrich) at room temperature for 15 min, and washed twice with PBS. Cells were permeabilized using 0.1% Triton-100 for 15 min at 4 °C and blocked with 5% horse serum. Primary antibodies were incubated with the cells at 4 °C for 16 h. After incubation, the cells were washed twice with PBST and conjugated with secondary antibodies. The primary antibodies used were those against PSD95 (1:200, ab18258), SYP (1:200, ab32127) and MYOG (1:500 ab124800) (All from Abcam, Cambridge, UK); PAX6 (1:100, 901301), NES (1:200, 841901) and TUBB3 (1:500, 801201) (All from BioLegend, San Diego, CA, USA); OCT4 (1:200, SC-8628), NCAD (1:500, SC-53488) and SOX1 (1:200, SC-17318) (All from Santa Cruz Biotechnology, Dallas, TX, USA); NF (1:500, MAB1592) and CHAT (1:100, AB144P) (All from Sigma-Aldrich); MHC (1:50, MF 20), TTN (1:40, 9D10), and HB9 (1:50; 81.5C10) (All from Developmental Studies Hybridoma Bank, DSHB, Iowa City, IA, USA); SV2A (1:100, PA5-52476) and MYOD (1:100, PA5-23078) (All from Invitrogen, Waltham, MA, USA); MHC (1:100, MAB4470) and NANOG (1:30, AF1997) (All from R&D Systems, Minneapolis, MN, USA); ISL1 (1:500; GT15051, Neuromics, Edina, MN, USA), OLIG2 (1:500; NBP1-28667, Novus Biologicals, Centennial, CO, USA). AChRs were labeled with α-bungarotoxin. Cell nuclei were stained with DAPI. Fluorescence images were captured using an upright microscope (Imager Z1, Zeiss, Oberkochen, Germany), upright fluorescence microscope (BX61VS, Olympus Corporation, Tokyo, Japan), and confocal microscope (ANDOR BC43, Oxford Instruments, Abingdon-on-Thames, Oxford, UK). The number, volume, intensity, and distribution of specific markers were quantified using images from three independent experiments on ImageJ (National Institutes of Health, Bethesda, MD, USA), Olympus CellSens Dimension Desktop 3.1, and IMARIS (Oxford Instruments).

### Stimulation of myotube contraction using CaCl_2_

NMJ cells were observed under an ECLIPSE Ti2-E microscope (Nikon, Tokyo, Japan), and videos were recorded for 30 s, after which, CaCl_2_ was added to the cell culture dish at 25 mM final concentration. SKM contractions were then recorded for 30–60 s. In some experiments, 200 μM curare (Sigma-Aldrich) was added to the MN–SKM coculture to inhibit NMJ activity. The intensity changes of ROIs were analyzed, and the contraction numbers were calculated using NIS-Elements AR (Nikon).

### Statistical analysis

All data were collected from three independent experimental replicates. Typical sample sizes were chosen in accordance with previous publications and are similar to those generally employed in the field. For 2D imaging, two images were obtained per replicate, totaling six images across the three replicates. For 3D confocal imaging, 1–3 images were collected per replicate, totaling 4–8 images across the three replicates. The ICC images and SKM contraction videos were randomized using random numbers. Subsequently, an independent investigator, who was blinded to the group assignments of the randomized images and videos, conducted the analysis. Randomization was not used in other experiments. Data are expressed as mean ± standard error of the mean. Statistical analysis was presented in each figure legend. The similarity of variance between groups was assessed, and statistical analyses were performed using an unpaired two-tailed Student’s *t*-test and one-way ANOVA followed by Tukey’s post hoc test. All calculations were performed using GraphPad Prism 8 software (GraphPad Software, Inc.). Statistical significance is indicated in figures (*p < 0.05; **p < 0.01; ***p < 0.001; and N.S., not significant).

## Supplementary information

Figure S1 Figure Legend

Video S1

Video S2

Video S3

Video S4

Video S5

Video S6

Video S7

Video S8

Video S9

Video S10

Video S11

Video S12

Video S13

## Data Availability

All relevant data are presented in the figures and supplementary figures. Any raw data and materials that can be shared will be released upon reasonable request via a material transfer agreement.
