# Supplementary material for: Rapid iPSC-derived neuromuscular junction model uncovers motor neuron dominance in amyotrophic lateral sclerosis cytopathy
Source: Cell Death Discov. 2025 Jan 25;11:23. doi: 10.1038/s41420-025-02302-5 (PMC11762734; doi:10.1038/s41420-025-02302-5)
Supplement: Supplementary file 1 — Figure S1 Figure Legend [file 41420_2025_2302_MOESM1_ESM.docx]

**Fig. S1**. **Negative controls for all primary antibodies used in immunocytochemistry (ICC).** **(A)** ICC staining of pluripotent stem cell markers OCT4 and NANOG; neural markers SYP, PSD95, PAX6, NCAD, SOX1, NF, TUBB3 and NES; myoblast and muscle cell markers MYOD, MYOG, MHC and TTN in undifferentiated iPSCs. **(B)** ICC staining of neural markers SOX1 and NF; motor neuron markers OLIG2, HB9, ISL1 and CHAT in iPSC derived non-motor neurons. Scale bars: 50 μm.
